# Hypertension in Children and Adolescents: A Position Statement From a Panel of Multidisciplinary Experts Coordinated by the French Society of Hypertension

**DOI:** 10.3389/fped.2021.680803

**Published:** 2021-07-07

**Authors:** Béatrice Bouhanick, Philippe Sosner, Karine Brochard, Claire Mounier-Véhier, Geneviève Plu-Bureau, Sébastien Hascoet, Bruno Ranchin, Christine Pietrement, Laetitia Martinerie, Jean Marc Boivin, Jean Pierre Fauvel, Justine Bacchetta

**Affiliations:** ^1^Service d'Hypertension Artérielle et Thérapeutique, CHU Rangueil, CERPOP, Université de Toulouse, Inserm, UPS, Toulouse, France; ^2^Centre Médico-Sportif MON STADE, Paris, France; ^3^Hôpital Hôtel-Dieu, APHP, Centre de Diagnostic et de Thérapeutique, Paris, France; ^4^Laboratoire MOVE (EA 6314), Université de Poitiers, Faculté des Sciences du Sport, Poitiers, France; ^5^Service de Néphrologie Médecine Interne Pédiatrique, Hôpital des Enfants, CHU Toulouse, Toulouse, France; ^6^Institut Cœur-Poumon, Médecine Vasculaire et HTA, CHU, Université Lille, EA 2694 - Santé Publique: Epidémiologie et Qualité des Soins Lille, Lille, France; ^7^Unité de Gynécologie Médicale, AP-HP, Hôpital Port-Royal, Université de Paris, Paris, France; ^8^Pôle des Cardiopathies Congénitales du Nouveau-Né à L'adulte - Centre Constitutif Cardiopathies Congénitales Complexes M3C, Groupe Hospitalier Paris Saint-Joseph, Hôpital Marie-Lannelongue, Inserm U999, Université Paris-Saclay, Le Plessis-Robinson, France; ^9^Centre de Référence des Maladies Rénales Rares, Service de Néphrologie Rhumatologie et Dermatologie Pédiatriques, Filières Maladies Rares ORKID et ERK-Net, Hospices Civils de Lyon, Hôpital Femme Mère Enfant, Bron, France; ^10^Faculté de Médecine Lyon Est, Université Lyon 1, Lyon, France; ^11^Unité de Néphrologie Pédiatrique, CHU Reims, Reims, France; ^12^Centre de Reference des Maladies Rares de la Croissance et du Développement, Université de Paris, Endocrinologie et Diabétologie Pédiatrique, AP-HP, Hôpital Robert-Debré, Paris, France; ^13^Département de Médecine Générale, Université de Lorraine, Inserm CIC-P Pierre Drouin Vandœuvre-Lès-Nancy, Vandœuvre-lès-Nancy, France; ^14^Service de Néphrologie Hospices Civils, Hôpital Edouard Herriot, Lyon, France; ^15^UMR CNRS 5558, Université Claude Bernard Lyon 1, Villeurbanne, France

**Keywords:** high blood pressure, French position statement, adolescents, children, hypertension

## Abstract

Hypertension is much less common in children than in adults. The group of experts decided to perform a review of the literature to draw up a position statement that could be used in everyday practice. The group rated recommendations using the GRADE approach. All children over the age of 3 years should have their blood pressure measured annually. Due to the lack of data on cardiovascular morbidity and mortality associated with blood pressure values, the definition of hypertension in children is a statistical value based on the normal distribution of blood pressure in the paediatric population, and children and adolescents are considered as having hypertension when their blood pressure is greater than or equal to the 95th percentile. Nevertheless, it is recommended to use normative blood pressure tables developed according to age, height and gender, to define hypertension. Measuring blood pressure in children can be technically challenging and several measurement methods are listed here. Regardless of the age of the child, it is recommended to carefully check for a secondary cause of hypertension as in 2/3 of cases it has a renal or cardiac origin. The care pathway and principles of the therapeutic strategy are described here.

## Introduction

Hypertension (HTN) is much less common in children than in adults, but the prevalence of HTN among children and adolescents is negligible: about 2.2% in the United States (US) of America (1). A third of newly diagnosed hypertensive children demonstrate significant target organ damage, left ventricular hypertrophy (LVH) and arterial stiffness in adulthood (2). Several studies demonstrated the evidence of blood pressure (BP) tracking from childhood into adulthood as childhood BP is associated with BP in later life (3).

Although US, Canadian and European guidelines for the management of paediatric HTN have been published for several decades, diagnosis and of the healthcare pathways of the HTN remain heterogeneous among practitioners (4–10).

Discrepancies between several US recommendations exist: The US Preventive Services Task Force stated that the current evidence is insufficient to screen for primary HTN in asymptomatic children and adolescents whereas, according to the potential implications of HTN on cardiovascular disease in adulthood, an early screening has been advocated by other US societies (4–6). At the moment, no French recommendations about the management of children and adolescent HTN were already published. The aim of the group of experts from the French Society of Hypertension was to perform a review of the literature drawing on the latest expert consensuses or international recommendations up to 2020. The aim of the resultant deliberately concise and practical position statement is to enable the document to be used in everyday practise.

## Methodology

The group of experts rated the recommendations using the GRADE approach, which rates both the overall certainty of the scientific evidence (number and quality of studies) and the strength of the resulting recommendations (strength of evidence in favour of/against the recommendation). The recommendations are rated in grades, i.e., Grade A (high scientific certainty in the body of evidence), Grade B (medium scientific certainty in the body of evidence), and Grade C (low scientific certainty in the body of evidence), and classes, i.e., class 1 (recommended), class 2 (suggested), and class 3 (not recommended). The document was read by several members of French scientific societies: the *Société de Cardiopédiatrie* (Society of Paediatric Cardiology), the *Société de Néphrologie pédiatrique* (Society of Paediatric Nephrology), and the *Société Française d'Endocrinologie et de Diabétologie Pédiatrique* (French Society of Paediatric Endocrinology and Diabetology). The following will not be addressed: malignant hypertension and neonatal hypertension. Furthermore, the principles of the therapeutic strategy and treatments will be discussed in general only, not in detail.

### Definition of HTN in Children and Adolescents

In the absence of data on cardiovascular morbidity and mortality associated with a certain level of BP, the definition of HTN in children is a statistical value based on the normal distribution of BP in the paediatric population. Children and adolescents are considered as having elevated BP (replaces the term prehypertension) when the measurement is between the 90th and 95th percentiles for age, height, and gender, and as having HTN when their BP is greater than or equal to the 95th percentile. Stage 1 HTN corresponds to a systolic BP (SBP)/diastolic BP (DBP) measurement ≥95th percentile and stage 2 HTN corresponds to a systolic or diastolic value ≥95th percentile +12 mm Hg. It is important that several measurements of BP be taken over time before HTN is diagnosed and these measurements should be made under correct conditions (see corresponding chapter).

BP curves in children take into account their gender, age and height. The curves used come from two main references: the auscultatory measurements published by the American Task Force in 2004 (11), and the German curves published in 2011 (12). It should be noted that the use of the American values was endorsed in 2016 by the European Society of Hypertension (7). These curves were also updated in 2017 by excluding BP measurements in overweight or obese children in order to avoid the bias related to the frequent association of HTN with overweight children (9). They therefore represent normal BP in children of normal weight, and are therefore more “stringent” than the former ones. However, since our goal here is to provide general paediatricians and general practitioners with practical tools to use in daily life, a simplified table is provided for the easy and daily identification of children in whom it is advisable to check BP several times to screen for possible HTN (Table 1). In case of clinical suspicion of HTN, websites that allow calculation of the BP percentile for age, height and gender are easily available, for example: The Baylor College of Medicine site: https://www.bcm.edu/bodycomplab/Flashapps/BPVAgeChartpage.html or The International Paediatric Hypertension Association site: http://www.iphapediatricHTN.org/resources/calculators/.

**Table 1:** Simplified blood pressure table for the screening of potential arterial hypertension.

**Age (years)**	**Blood pressure (mm Hg)**			
	**Boys**		**Girls**	
	**SBP**	**DBP**	**SBP**	**DBP**
1	94	49	97	52
2	97	54	98	57
3	100	59	100	61
4	102	62	101	64
5	104	65	103	66
6	105	68	104	68
7	106	70	106	69
8	107	71	108	71
9	109	72	110	72
10	111	73	112	73
11	113	74	114	74
12	115	74	116	75
13	117	75	117	76
14	120	75	119	77
15	122	76	120	78
16	125	78	121	78
17	127	80	122	78

*This table provides the 90th percentile of blood pressure for age in a child at the 5th percentile for height. This table is derived from the nomograms of the Fourth Report on the Diagnosis, Evaluation, and Treatment of High Blood Pressure in Children and Adolescents (11)*.

### Recommendation No. 1

To define HTN, we recommend using the normative BP tables developed according to age, height, and gender, a simplified version of which is provided in Table 1 (Grade C class 1).

### Epidemiology

The prevalence of HTN and prehypertension (more often referred to as elevated BP) in school-aged children (8–17 years) has increased significantly since 1988, as shown notably in the American National Health and Nutrition Examination Survey (NHANES) epidemiological studies (13). It is actually very complex to assess the prevalence of HTN in children and adolescents: based on the normative BP values of 2004 (11), studies in 2007 estimated the prevalence of HTN to be between 2 and 4% in a population of school-aged children and adolescents, but the value is probably overestimated (14, 15). Other studies carried out in American populations gave percentages that were ten times lower, between 0.1 and 0.3%, and probably closer to the values observed in France, even if figures are lacking (16, 17). The prevalence of elevated BP (defined by a BP >90th percentile or >120/80 mm Hg and <95th percentile) appears to waver between 2.2 and 3.5%. The figure increases in overweight and obese individuals (14, 15). Tu et al. (18) confirmed in a population of 1,111 children that a parallel relationship exists between an increase in body mass index (BMI) and an increase in BP. With a BMI <85th percentile, adiposity has little impact on BP. However, the risk of developing high BP or HTN is increased by a factor of 4 in overweight children, even before the stage of obesity (BMI between the 85th and 94th percentiles), regardless of the child's age. The American Academy of Paediatrics (AAP), backed by the American Heart Association (AHA), revised the 2004 guidelines in 2017 (9). Blood pressure measurements of overweight or obese children were excluded from the previous tables given the close link between being overweight and obesity and elevated BP-HTN, and for adolescents aged 13 years or more, the recommendations of the AAP were aligned with those for adults. Based on these new recommendations, the prevalence of high BP was found to have increased by 1.5% in a recent American study including 22,224 students aged 10–17 years, but the prevalence of HTN remained between 2 and 4% (19). A recent study from Greece highlighted an alarming proportion of almost 25% of school-children aged 9–13 years, the presence of hypertension being positively associated with body mass index and waist circumference in both genders, and with sedentary behaviours only in boys (20); with the recent COVID crisis and shut-down policies inducing more sedentarity and more obesity, this effect may even become more relevant. Consequently, a higher proportion of left ventricular hypertrophy, an independent cardiovascular risk factor, may therefore be detected (21). In a study comparing the impact of two different guidelines (9, 11), the proportion of participants with an abnormal left ventricular mass categorised as hypertensive significantly increased from 20 to 31% as defined in the Fourth Report and CPG, respectively (21). Another study performed in 951 individuals referred to a Paediatric Center for Cardiovascular Risk Prevention showed a 12% increase in the prevalence of children with BP above the 90th percentile using the most recent normograms (9, 22); moreover, the application of more 'physiological' nomograms, based on a population of normal-weight children, did not yield any advantage in identifying individuals with early cardiac organ damage (22). As such, the gain in sensitivity to detect cardiac hypertrophy is counter-balanced by the loss in specificity.

Childhood obesity as well as an increased prevalence of elevated BP are two risk factors for premature cardiovascular disease in adults that should be screened for and treated at an early stage (23, 24).

### Frequency of Secondary HTN vs. Essential HTN in Paediatrics

In an American study conducted in Texas between 2005 and 2011 in 275 hypertensive children, 43% had essential HTN and 57% had secondary HTN, but the population was biassed since it consisted of hospitalised patients (25). Primary or essential HTN is the predominant form of HTN in 6–12 year-olds and adolescents, especially in those with a family history of HTN or who are overweight and/or obese. Given the prevalence of obesity in the USA, these American data probably overestimate the frequency of essential HTN in French children and adolescents. In general populations, it is estimated that <10% of all paediatric patients display secondary HTN (26), but secondary HTN is more prevalent in younger children, especially in those under 6 years of age (25, 27). In these cases, secondary HTN is primarily caused by renal and/or renovascular disorders, which account for 63 to 74% of the cases.

### When to Measure BP

In children under the age of 3 years, BP should be checked regularly in the following cases: history of low birth weight <2,500 grammes, kidney disease or uropathy, congenital heart disease (aortic coarctation the most), solid organ or bone marrow transplantation, intracranial hypertension, treatment with medicines known to cause HTN, systemic diseases which may be complicated by HTN (neurofibromatosis, tuberous sclerosis, etc.), and syndromes that may be associated with HTN (Williams-Beuren syndrome, etc.) (11). It should be noted that measuring BP in children <3 years of age can be technically very challenging because of cuff size constraints and the restlessness of children (that is frequent…): it can sometimes take up to 30 or 40 min to obtain an accurate BP measurement.

From the age of 3 years, HTN is often asymptomatic in children like in adults and, therefore, it appears justified to measure BP systematically on an annual basis in the same way as weight, height and BMI, or at each medical visit if the child has a personal history of nephropathy, diabetes mellitus, dyslipidaemia, being overweight/obesity, taking treatments that may induce HTN or a parental history of HTN or early-onset coronary artery disease (men <55 years old and women <65 years old) (9, 11).

### Recommendation No. 2

a) Before the age of 3 years, we recommend to measure BP systematically in the following cases:
  - History of low birth weight <2,500 grammes;
  - Kidney disease or urological abnormality;
  - Congenital heart disease;
  - Solid organ or bone marrow transplantation;
  - Intracranial HTN;
  - Exposure to medicines known to cause HTN;
  - Systemic disease which may be complicated by HTN (neurofibromatosis, tuberous sclerosis, etc.) (Grade C class 1).
b) After the age of 3 years, we recommend to measure BP systematically at least once a year in the same way as weight, height and BMI as HTN is most often asymptomatic (Grade C class 1).

### Method of BP Measurement: Office or Clinical BP, Ambulatory Blood Pressure Monitoring (ABPM), Home Blood Pressure Monitoring (HBPM)

BP is measured differently according to the age of the child:

In new-borns (0–1 month), the gold standard is intra-arterial measurement (28).

After 1 month and up to 3 years of age, the method of reference is non-invasive measurement using an aneroid sphygmomanometer (alternative to mercury, which is not authorised), with palpation of the radial pulse and auscultation of the antecubital fossa (elbow crease).

From 3 to 12 years of age, European (7, 29) and North-American (9, 30) guidelines agree on BP measurement in the office or a so-called “clinical” setting, using the auscultatory manual method and an aneroid sphygmomanometer (Grade C). In the event of an abnormal BP measurement using an automated oscillometric device (which tends to overestimate BP), the BP measurement should be checked using the auscultatory manual method (Grade C) (7, 9, 28–30).

From 13 to 17 years, European (7, 29) and North-American (9, 30) guidelines agree that BP monitoring should be similar to that of adults with a BP measurement being taken in a clinical setting and completed by 24-h ambulatory BP monitoring (ABPM) or home BP monitoring (HBPM), here too with a cuff adapted to the size of the arm and using a validated automatic device (7, 9, 29, 30).

The following conditions must be respected as much as possible when clinically measuring BP:

The environment: it must be calm, with no talking [talking during measurement may cause a deviation of +10 mm Hg in SBP and DBP (31)]; the effect of external factors must be minimised by patients getting enough sleep and avoiding consumption in the previous 24 h of very salty foods or drinks with a high caffeine content, and even tobacco;

Position of the child: seated for 5 min with feet placed on the ground and not suspended (32); the child's legs must not be crossed, his/her arm must be bared, and his/her back and arm must be supported with the antecubital fossa at the level of the heart (30, 33) [a cuff placed on a garment can cause a SBP deviation of +5 to 50 mm Hg, an unsupported back a deviation of +6 to 10 mm Hg, and an unsupported arm a deviation of + 1–7 mm Hg for SBP and 5–11 mm Hg for DBP (31)];

Equipment: a suitably sized cuff must be used [cuff bladder width at least 40% of the circumference of the arm and length covering 80 to 100% of circumference of the arm (30); too small a cuff may result in falsely high readings (11): +10 mm Hg for SBP, +2 to 8 mm Hg for DBP (31), and too wide cuff, falsely low readings (11)].

Measurement: the right arm is the preferred site [site spared in the event of coarctation of the aorta which would result in underestimation of the measurement in the left arm depending on its location (11, 30)]; the stethoscope must be placed over the antecubital fossa, the cuff must not be excessively inflated [limit: 30 mm Hg above the level of disappearance of the radial pulse) (30); a cuff that is too tight can cause restlessness in children (31)]; SBP corresponds to the reappearance of blood flow (Korotkoff phase I); DBP usually corresponds to Korotkoff phase IV (muffling of sounds) which is used instead of phase V (disappearance of sounds) because often in children, Korotkoff sounds are perceived up to 0 mm Hg; The auscultatory method produces measurements with an accuracy of 2 mm Hg, while automatic devices give results with an accuracy of 1 mm Hg (30).

Aneroid sphygmomanometers should be calibrated on a semi-annual basis (11), and automatic devices according to manufacturer recommendations (34). In all cases, the BP monitors must have been validated in children (Grade C). Few automatic oscillometric devices have been validated in children; they cannot distinguish between Korotkoff phases IV and V for DBP. Regarding wrist monitors, they have not been validated in children in any large studies and should therefore be avoided (32). A list of validated devices has been drawn up by Stergiou et al. (29). For the diagnosis of HTN in the event of elevated BP during a 1st visit in asymptomatic patients, measurements must be repeated during 2 other visits 1 month apart (Grade C), or closer together in high-risk cases (Grade B) (30). In some cases, HTN in children is a medical emergency (catecholamine associated hypertension, acute kidney injury, intracranial haemorrhage, neuroblastoma…) and in case of severe HTN, measurements do not need to be repeated.

Even though they are useful tools, ABPM or HBPM [which reference values in paediatrics derive from a single study in a relatively small population (35)] should be used only in selected situations by experts in paediatric HTN, on a case-by-case basis:

Twenty four hour ABPM may be prescribed by experts for paediatric HTN, preferably in children aged 5 years or older and measuring at least 120 cm as it is interpreted with appropriate paediatric normative data for children 5 years of age or height 120 cm (34), and performed with a validated device at their homes (Grade C). This type of monitoring can be indicated to confirm the diagnosis of HTN, before treatment is initiated (29), and all the more so if the cardiovascular risk is high (Grade B), to analyse the day/night rhythm, or if a white-coat effect is suspected (9). It may also be indicated when the response to treatment is insufficient (Grade B) (9). However, 24 h ABPM may not be well-tolerated and requires the active cooperation of the whole family (36). Its reproducibility is higher than HBPM, but references are lacking for children <120 cm in height (28). A schema for the classification of HTN in children is presented in Table 2 (37).

**Table 2:** Suggested schema to classify blood pressure in children (34).

**Classification**	**Office BP**	**Mean ambulatory SBP or DBP during awake or sleep period, or both**	**SBP or DBP load (%)**
White coat hypertension	≥95th percentile	<95th percentile	<25
Masked hypertension	<95th percentile	≥95th percentile	≥25
Ambulatory hypertension	≥95th percentile	≥95th percentile	25–50
Severe ambulatory hypertension	≥95th percentile	≥95th percentile	>50

*BP, blood pressure; DBP, diastolic BP; SBP, systolic BP*.

HBPM, requires more studies before it can be used for the diagnosis of HTN in children (38), but it could be a useful backup tool for the monitoring of known HTN (Grade C) (9), particularly in older children and adolescents.

The list of oscillometric devices validated in paediatric age are available online (www.dableducational.org).

### Recommendation No. 3

a) In children, we recommend to measure BP using an auscultatory method and an aneroid sphygmomanometer (Grade C, class 1).
b) In the event of an abnormal BP reading with an oscillometric BP monitor (which tends to overestimate BP), we recommend to check the measurement using an auscultatory manual method (Grade C, class 1).
c) We recommend to measure BP in a calm place in a child who has been sitting for 5 min with his/her feet on the floor (not suspended), whose back and arms are supported, and whose antecubital fossa is at heart level. An appropriately sized cuff and a BP monitor that has been validated in children must be used (Grade C, class 1).
d) We recommend to measure BP in the right arm (site spared in case of coarctation of the aorta) (Grade C, class 1).
e) In the event of elevated BP during a 1st visit, we recommend in asymptomatic patients to repeat measurements during 2 other visits 1 month apart (Grade C, class 1) or closer together in high-risk cases (Grade B, class 1).
f) 24-h Ambulatory Blood Pressure Monitoring (ABPM) can be performed in selected situations by experts in paediatric HTN, on a case-by-case basis (Grade B, class 1) for children >120 cm in height, but it may not be well-tolerated; ABPM is not recommended in children who measure <120 cm.
g) We recommend to only use Home Blood Pressure Monitoring (HBPM) for the monitoring of known HTN (Grade C, class 1) given the lack of reference values for the diagnosis of HTN.

### Clinical Examination

The diagnostic assessment of HTN in a child must be carried out in two stages including the recording of the child's medical past and the performance of a clinical examination followed by laboratory tests and a radiological evaluation.

The aims of a complete physical examination are to provide clues to potential secondary causes of HTN and assess possible hypertensive end organ damage (9). Then, the purpose of the clinical interview is to try and determine whether the HTN is recent or long-standing, to describe the neonatal period (IUGR, prematurity, history of umbilical artery catheterization which increases the risk of secondary vascular stenosis), and to check for a family or personal history of neurological diseases (neurocutaneous disorders), acquired or hereditary kidney disorders, an inherited form of HTN (Liddle's syndrome), and hereditary paraganglioma-pheochromocytoma. A comprehensive list must also be drawn up of current or recent treatments (sympathomimetic drugs, prednisone, calcineurin inhibitors, fludrocortisone, oral contraceptives, etc.). Enquiries should be made about possible excessive consumption of liquorice, mercury poisoning, sudden and brief changes in skin colour (pallor or redness), sudden agitation, throbbing headaches associated with tachycardia (palpitations), profuse sweating suggestive of pheochromocytoma, or exertional dyspnoea suggestive of heart failure and of coarctation of the aorta.

The clinical examination should begin with the taking of measurements, i.e., weight, height and BMI with analysis of the growth curves of the French Auxology Group, and an assessment of the Tanner pubertal stage. Cardiac examination (murmurs, heart failure using Ross/NYHA functional class), abdominal examination (tumour, renal arteries bruits, and pulses of aorta) and neurological examination (intracranial hypertension) must be checked. The superficial arteries should then be palpated and auscultated for pulse discrepancies and an increase in the differential BP value between the arms and legs suggestive of coarctation of the aorta. The examiner must then check whether the patient presents absent or weak superficial pulses, and carotid, aortic abdominal, renal or femoral bruits suggestive of vascular stenosis, and cardiac erethism suggestive of hyperthyroidism or pheochromocytoma. The patient's skin must be examined for café au lait spots (neurofibromatosis type 1), achromic naevus with sebaceous adenomas (tuberous sclerosis), angiomas (Von Hippel-Lindau disease), pseudoxanthoma elasticum in the large flexor surfaces, or signs of acrodynia. Dysmorphic features should be noted: the moon face of Cushing's syndrome, the elfin-like facial features of Williams-Beuren syndrome, the stunted growth and facial dysmorphia of Turner's syndrome, the arachnid morphology associated with Marfan syndrome. Abnormal tallness may be suggestive of acromegaly. Large palpable kidneys may be suggestive of autosomal recessive or dominant polycystic kidney disease. A goitre, especially if associated with a thrill (vibration in hand on palpation) and a murmur on auscultation and/or exophthalmos suggests hyperthyroidism. Neuroblastomas and nephroblastomas may be revealed by HTN caused by secretion or compression. Table 3 summarises the diagnostic clinical approach when elevated BP or HTN is observed.

**Table 3:** Clinical examination in a child or adolescent with elevated blood pressure or hypertension.

**- Height, weight and BMI**
**- Morphological examination:**
^*^ Check for dysmorphic facial features (Turner's syndrome, elfin-like facial features of Williams-Beuren syndrome, Alagille syndrome, etc.);
^*^ Café au lait spots (neurofibromatosis), sebaceous adenomas (tuberous sclerosis), angiomas (Von Hippel-Lindau disease);
^*^ Check for arachnid morphology and joint hypermobility (Marfan syndrome) and/or pseudoxanthoma elasticum;
^*^ Check for exophthalmos, for a goitre; (hyperthyroidism);
^*^ Check for the moon face of Cushing's syndrome, stretch marks (hypercortisolism)
**- Cardiovascular examination:**
^*^ Measure BP in the 4 limbs;
^*^ Check for and auscultate superficial pulses (coarctation of the aorta);
^*^ Check for a heart murmur and abdominal (renal artery stenosis), carotid and femoral bruits;
^*^ Check for signs of heart failure.
**- Abdominal examination**
^*^ Check for masses (Wilms' tumour, neuroblastoma, autosomal dominant or recessive polycystic kidney disease);
^*^ Check for hepatosplenomegaly (autosomal recessive polycystic kidney disease).
**- Neurological examination with an ocular fundus (Alagille syndrome)**.

*BP, blood pressure*.

### Recommendation No. 4

In children and adolescents being evaluated for high BP, the practitioner should perform a physical examination to identify findings suggestive of secondary causes of HTN listed in Table 3 (Grade B, class 1).

### Additional Tests

The recommendation to perform additional tests is not based on any studies but on expert opinions in consensus guidelines, the most recent of which are listed here (9).

The following tests should be performed in all children and adolescents regardless of the results of the clinical examination: blood electrolytes (serum potassium), serum creatinine (calculation of estimated glomerular filtration rate using the 2009 bedside Schwartz formula); urine sediment examination of the first morning urine; quantitative estimation of cells in urine (haematuria); urine protein to creatinine ratio (normal: <50 mg/mmol before 2 years of age and <20 mg/mmol after 2 years of age).

The following tests should also be requested in overweight or obese children or adolescents (BMI >95th percentile) and in those with a family history of dyslipidaemia: AST, ALT; fasting total cholesterol, HDL, LDL and triglycerides; fasting blood sugar.

Some tests remain optional and depend on the individual's medical history, the physical exam and the initial additional tests. They may be requested by a paediatric cardiologist, paediatric nephrologist or paediatric endocrinologist: TSH, drug screening (in adolescents), blood count, serum calcium, plasma renin and aldosterone assay between 8 and 10 a.m. without interfering treatments, methoxyamine in 24-h urine or plasma (plasma not reimbursed in France in non-hospital care), 24-h cortisol urine test, serum toxin assays: lead, cadmium and mercury.

Toxic causes may be suspected in certain specific situations, particularly lead poisoning. A correlation was found between high blood lead levels and increased BP in a study on 122 children from underprivileged populations, but without matching for low socioeconomic status which was a confounding factor (39). Cadmium is potentially nephrotoxic in children but the relationship with the BP level has not been demonstrated (40). Acute mercury poisoning in children is associated with BP surges like those observed in pheochromocytoma (41, 42). Finally, neonatal exposure to phthalates may be associated with higher BP values in childhood, but not with the onset of HTN in adulthood, and several of their components are currently being studied (43). Assaying them is therefore premature (9).

Testing for microalbuminuria in case of essential HTN is probably not helpful. Its value is not as backed as in adults in the literature because it may be a marker of kidney injury, or be linked to insulin resistance or obesity, and can even occur in the absence of HTN, for example in children having undertaken physical activity. Its prognostic significance is not known. Nevertheless, in paediatrics, there are many knowledge gaps and the care is often based on adult experience. The existence of albuminuria in children, although not proven, suggests choosing angiotensin-converting-enzyme inhibitors (ACEi)/angiotensin receptor blockers (ARBs) as first line treatment in the absence of contraindications. In European and Canadian recommendations, microalbuminuria or albumin-to-creatinine ratio (first-morning measurement) is recommended for routine clinical use for the assessment of target organ damage in all children with HTN whereas for US recommendations, routine testing for microalbuminuria is not recommended for children and adolescents with primary HTN (7, 9, 37).

Testing for endocrine HTN such as that linked to a block (enzyme deficiency, the most frequent of which is 11β hydroxylase), an adrenal adenoma, or pituitary hypersecretion requires the knowledge of a specialist. Endocrine causes account for 0.05 to 6% of cases of secondary HTN (25, 27, 44, 45). They call for a careful clinical interview and examination.

#### Other Tests

ECG is used fairly systematically after 12 years of age as, before that age, it requires interpretation by an experienced paediatric cardiologist (physiological T-wave inversion in children…). It can be used to eliminate arrhythmia in case of hypercalcaemia or abnormal potassium levels. It should be noted that ECG is a poor diagnostic tool for the detection of LVH with a very low positive predictive value. Cardiac ultrasound should therefore be the method of choice for the detection of LVH in children (9, 46).

Echocardiography should be performed systematically to screen for coarctation of the aorta, check for LVH as, unlike ECG, it's predictive value for identifying LVH is high, to measure the left ventricular mass index (although indexation based on a study of normotensive children may be questionable) and interventricular septal thickness correlated with BP; to measure fractional shortening which can be altered in acute hypertensive episodes. Testing for LVH is indicated in children in the assessment of target organ damage and in case of pharmacological treatment of HTN (9). Repeated testing as part of monitoring of an abnormality or pharmacological treatment should be approved by a paediatric cardiologist or cardiologist.

An exercise test should be considered in the child to evaluate symptoms that are triggered or aggravated by exercise; to assess the response to exercise when a cardiac pathology exists (congenital or acquired), including ischemia or arrhythmia; or to evaluate the efficacy of medical or surgical treatments (47).

A cardiopulmonary exercise test should be considered in the child with a congenital heart disease: to assess prognosis; to evaluate the patient's functional capacity and prescribe an adapted physical activity programme; or before a cardiac rehabilitation programme (47).

Kidney ultrasound must be performed systematically to screen for underlying uropathy, renal hypoplasia, or discrepant kidney sizes. The size of the kidneys should be mentioned on the report (kidney size charts according to age), as well as the ultrasound appearance of the parenchyma and cortical thickness.

Doppler ultrasound of the renal arteries may be requested as part of a specialist assessment and must be performed by a paediatric radiologist or an experienced angiologist in children over 8 years of age who will cooperate with the procedure and on a case-by-case basis in younger children who are suspected of having renovascular HTN.

The tests for identification of renovascular HTN need to be prescribed by a specialist. There are no precise criteria for its identification: some experts suggest it should be looked for in children and adolescents with stage 2 HTN, in those with predominantly diastolic HTN, particularly in ambulatory measurements, in those with hypokalaemia, abnormal renal function; urine sediment abnormalities or those with discrepant kidney sizes on ultrasound (9). The sensitivity and specificity of renal artery Doppler ultrasound was found to be, respectively, 64–90 and 68–89% in 2 series, with the best results being obtained in non-obese and cooperative children over 8 years of age (48, 49). Doppler ultrasound of the renal arteries is fairly systematically requested in usual practise because it is non-invasive and non-irradiating.

Deciding whether renal artery computerised tomography (CT-scan) or renal magnetic resonance imaging (MRI scan) is indicated requires specialist assessment and test feasibility (children who can't keep still), irradiation and renal function must all be put in the balance (9, 49). A discussion in a multidisciplinary team meeting on the diagnostic strategy is sometimes required.

There is practically no indication for ACE-inhibited renography that has only been incompletely assessed in the field for the screening of renovascular HTN in children and adolescents. It should be avoided except for specific indications targeted by a paediatric nephrologist (9).

Tests dedicated to evaluation of vascular structure or function (pulse wave velocity, carotid intima-media thickness) are still in the research stage and have not been sufficiently assessed to be recommended in routine clinical practise (7, 9).

Vascular ultrasound of the aorta may be requested as part of a specialist assessment. Coarctation may be suggested in cases of a pressure gradient and treatment-resistant HTN, whether or not associated with syndromes such as Turner syndrome, neurofibromatosis type 1, Takayasu disease, Williams-Beuren syndrome, or Alagille syndrome.

Children with snoring (≥3 nights per week), daytime fatigue, sleep enuresis (especially secondary enuresis), tonsillar hypertrophy or deficit/hyperactivity disorder may have obstructive sleep apnoea syndrome (OSAS) with consequent HTN (50). They should undergo polysomnography and the ABPM is the recommended method for assessing BP as both nighttime and daytime BP is affected by it. However, whether OSAS treatment results in improved BP in children is not known (51).

Genetic testing may be requested as part of a specialist assessment, for example in case of pheochromocytoma, but also in case of suspicion of Liddle's syndrome (Table 3).

### Recommendation No. 5

a) We recommend to perform the following tests in all children and adolescents regardless of the results of the clinical examinations: blood electrolytes (serum potassium), serum creatinine, assessment of glomerular filtration (using the Schwartz formula in children), urine sediment examination of the first morning urine (haematuria), urine protein to creatinine ratio (normal <50 mg/mmol before 2 years of age and <20 mg/mmol after 2 years of age) (Grade A, class 1).
b) The following tests should also be requested in overweight or obese children or adolescents (BMI >95th percentile) and in those with a family history of dyslipidaemia: fasting blood sugar, fasting lipid profile including total cholesterol, HDL and LDL, triglycerides, AST, and ALT (Grade A, class 1).
c) Once these initial examinations have been requested, we recommend to seek the opinion of a paediatric cardiologist and/or nephrologist and/or endocrinologist (Grade C, class 1).
d) We recommend to seek the expertise of a skilled paediatric cardiologist to interpret ECGs in children under 12 years of age (Grade B, class 1).
e) We recommend to systematically perform cardiac echocardiography to screen for LVH and isthmic coarctation of the aorta (Grade A, class 1).
f) We recommend to systematically perform Doppler ultrasound and kidney ultrasound to determine whether HTN can be attributed to a renal cause (asymmetry, renal hypoplasia) (Grade A, class 1).

### Secondary Causes of Hypertension

In young children (<6 years of age), HTN is most often secondary to another medical condition (27). In such cases, it is often symptomatic, but it may also be the clinical expression of other diseases (52) (Table 4).

**Table 4:** Main causes of secondary hypertension in paediatric patients.

**Renin excess (note that renin levels may be normal in patient sera)**
Nephropathies
Renovascular diseases with or without midaortic involvement
Isthmic coarctation of the aorta
Reninoma
**Primary catecholamine excess**
Paragangliomas and pheochromocytomas
Neuroblastomas
Hyperthyroidism
Mercury poisoning
**Primary mineralocorticoid (aldosterone, DOC) excess**
Conn's syndrome
Glucocorticoid-suppressible HTN
Adrenal enzyme block (11β-hydroxylase, 17α-hydroxylase)
Treatment with 9-α-fluorocortisol
**Increased tubular reabsorption of sodium**
Liddle's syndrome (epithelial sodium channel)
Gordon's syndrome (With No K kinase 1)
**Excessive activation of mineralocorticoid receptor by glucocorticoids**
Apparent mineralocorticoid excess syndrome (11β-hydroxysteroid dehydrogenase type 2)
Liquorice poisoning
Glucocorticoid resistance (mutation of the glucocorticoid receptor)
**Primary glucocorticoid excess**
Treatment with prednisone
Adrenal carcinoma
ACTH (adrenocorticotrophin) secreting adenoma
**Miscellaneous**
Neurological diseases: intracranial HTN, familial dysautonomia
Metabolic disorders: hypercapnia, hypercalcaemia
Medications (calcineurin inhibitors, oral contraceptives, etc.) and psychotropic drugs (amphetamine, cocaine)

The most frequent causes in 2/3 of cases are renal parenchymal diseases (especially glomerulopathies) and isolated renovascular diseases (fibromuscular dysplasia, diffuse coronary artery calcification); other causes include various syndromes (Williams-Beuren syndrome, Marfan syndrome, Alagille syndrome, Turner syndrome), neurocutaneous disorders (neurofibromatosis, Von Hippel-Lindau disease, tuberous sclerosis), pseudoxanthoma elasticum or vasculitis (Takayasu, periarteritis nodosa) and, in newborns, thrombosis of the renal artery (following umbilical catheter placement).

Coarctation of the aorta is the most common cause of HTN in new-borns (0–1 months) and infants (1–12 months). It sometimes occurs as part of a syndrome (Turner syndrome, Williams-Beuren syndrome). It is manifested by pulse and BP discrepancies between the arms and legs of more than 20 mm Hg, and by systolic murmur along the left sternal border with dorsal irradiation. In new-borns, coarctation of the aorta can manifest as cardiogenic shock when the ductus arteriosus closes. Antenatal echocardiography can suggest a risk of neonatal aortic coarctation, in particular as a result of the asymmetrical size of the heart chambers.

Endocrine causes, reported with a frequency of 0.05 to 6% according to the authors, are mainly represented by paragangliomas and pheochromocytomas, manifested by vasomotor episodes (sweating, headaches and palpitations); a genetic cause is identified in 25% of cases (multiple endocrine neoplasia type 2A or 2B, neurofibromatosis type 1, Von Hippel-Lindau disease, hereditary paragangliomas).

Mercury and lead poisoning may give rise to HTN as may some medicinal products such as corticosteroids, calcineurin inhibitors, oral contraceptives and even vasoconstrictors, but also psychotropic drugs such as cocaine and amphetamines. Overconsumption of liquorice should also be considered. Careful questioning should focus on obtaining a description of the hypertensive child's environment and medications.

Neurological (encephalitis and intracranial hypertension) and metabolic (hypercalcaemia, porphyria) causes are rarer.

Monogenic HTN is even rarer. It should be suspected in cases of collapsed plasma renin activity and a high aldosterone/renin ratio, especially if there is a family history of early-onset HTN (53).

The aetiologies can also be described according to their pathophysiological mechanisms (54): renin excess, primary catecholamine excess, primary mineralocorticoid (aldosterone) excess, excessive tubular reabsorption of sodium, primary glucocorticoid excess.

### Recommendation No. 6

a) Regardless of the age of the child, we recommend to carefully check for a secondary cause of HTN (Grade B, class 1).
b) We suggest focusing the search for secondary HTN on renal or cardiac causes as they account for 2/3 of the causes of secondary HTN (Grade B, class 2).

### The Healthcare Pathway

We remind here that urgent care of hypertensive crisis are not included in this review. Hypertension in children and adolescents is usually first diagnosed by the attending primary care physician. Even if all subspecialists should be able to perform the first line check-up for paediatric hypertension, recourse to a second line specialist could be required and it depends on local resources. As much as possible, patients should be referred to a paediatrician, whether a paediatric nephrologist, a paediatric cardiologist or a paediatric endocrinologist, or to corresponding adult specialists for adolescents, even if the patient may subsequently be referred from one specialist to another.

#### Who Should Be Addressed Indifferently to a Cardiologist or Paediatric Cardiologist or a Nephrologist or Paediatric Nephrologist or an Endocrinologist or Paediatric Endocrinologist?

Children or adolescents with a BP surge; those suspected of having a secreting pheochromocytoma or paraganglioma; those with hypokalaemia (primary or secondary hyperaldosteronism); those with a concomitant medical condition: neurofibromatosis type 1, dysplasia, Williams-Beuren syndrome, Alagille syndrome; those requiring pharmacological treatment.

#### Who Should Be Addressed More Specifically to a Nephrologist or Paediatric Nephrologist?

Children or adolescents with a family history of kidney disease (kidney failure, renovascular dysplasia, acute pyelonephritis), a history of uropathy, tubulopathy, kidney failure (evaluation of glomerular filtration using the Schwartz formula), urine sediment disorders; proteinuria, vascular murmur in the renal area; asymmetric kidney size or a single kidney.

#### Who Should Be Addressed More Specifically to a Cardiologist or Paediatric Cardiologist?

Children or adolescents with clinical signs such as tachycardia, malaise, or heart murmur on auscultation; absent or weak pulse in the legs which can point to aortic coarctation; heart failure; a family history of heart disease; Williams-Beuren syndrome or Turner or Alagille syndromes which predispose to the risk of aortic coarctation.

#### Who Should Be Addressed More Specifically to an Endocrinologist or Paediatric Endocrinologist?

Children or adolescents with clinical signs of hypercortisolism (weight gain and growth failure, facial and truncal obesity, proximal amyotrophy, vertical purple stretch marks, facial erythrosis and hirsutism); short stature (Turner syndrome) or abnormal tallness (acromegaly); a goitre; severe obesity.

### Recommendation No. 7

a) After a first diagnosis of HTN generally made by the attending physician or the paediatrician, we suggest to refer children or adolescents more specifically to a paediatric nephrologist or nephrologist when they present with (Grade C, class 2):
  - Family history of kidney disease (kidney failure, renovascular dysplasia, acute pyelonephritis);
  - History of uropathy, tubulopathy;
  - Kidney failure, urine sediment disorders; proteinuria
  - Vascular murmur in the renal area;
  - Asymmetric kidney size or a single kidney.
b) We suggest to refer children or adolescents more specifically to a paediatric cardiologist or cardiologist when they present with (Grade B, class 2):
  - Clinical signs such as tachycardia, malaise, or heart murmur on auscultation;
  - Absence or weak pulse in the legs which suggests aortic coarctation;
  - Family history of heart disease;
  - Williams-Beuren syndrome or Turner or Alagille syndromes which predispose to the risk of aortic coarctation.
c) We suggest to refer children or adolescents more specifically to a paediatric endocrinologist or endocrinologist when they present with (Grade B, class 2):
  - Clinical signs of hypercortisolism (weight gain and growth failure, facial and truncal obesity, proximal amyotrophy, vertical purple stretch marks, facial erythrosis +/– hirsutism);
  - Short stature (Turner syndrome) or abnormal tallness (acromegaly);
  - Goitre;
  - Severe obesity.

### Principles of the Therapeutic Strategy

#### Lifestyle Recommendations to Reduce High BP Values

Changing lifestyle and dietary habits is effective for all cases of HTN in children, even severe HTN. The early control of cardiovascular risk factors has been shown to be beneficial in terms of morbidity and mortality in adulthood. Overweight is probably the most important of the conditions associated with elevated BP in childhood (55). According to ESH recommendations, the goal is to maintain or achieve BMI <85th and life style measures should not only precede but also accompany pharmacological treatment (7).

Many paediatric guidelines have been emphasised the benefit of physical activity on aerobic fitness and mental health, physical rehabilitation and patient therapeutic education and a summary of the goals is recorded here. Moderate to vigorous physical aerobic activity, 40 min, 3–5 days/week is recommended avoiding more than 2 h daily of sedentary activities. Competitive sports should be limited only in the presence of uncontrolled stage 2 hypertension.

Fruits, vegetables and grain products should be preferred to sugar, soft drinks in excess and saturated fats. In the event of a water/salt overload, a strict low-sodium diet (0.3–0.5 mmol/kg/day) should be adopted. Less strict restriction (1 mmol/kg/day) is required in the event of severe HTN (severe glomerulonephritis, polycystic kidney disease). Once BP is controlled, sodium intake should remain moderate (2–3 mmol/kg/d).

Exposure to tobacco is a major risk factor for cardiovascular morbidity and prevention should begin during pregnancy (maternal smoking should be discouraged) and continued at all ages with patients being advised not to start smoking and encouraged to quit if necessary. Specific paediatric studies have already demonstrated the deleterious effect of tobacco exposure on vascular parameters as early as during teenage (56).

This behavioural change process involves parents and families and realistic goals are needed to improve adherence to the advices.

#### Pharmacological Treatment

Pharmacological treatment is indicated, regardless of the cause, in case of persistent HTN despite a change in lifestyle and dietary habits, and immediately in symptomatic patients or in those with stage 2 HTN, chronic kidney disease (CKD) or diabetes. The objective is to obtain a controlled BP lower than the 90th percentile for age (American recommendations) bearing in mind that European recommendations are even more stringent, with a controlled BP objective below the 75th percentile in the absence of kidney failure without proteinuria and below the 50th percentile for age in case of kidney failure and/or concomitant proteinuria (7).

Pharmacological intervention in children <6 years of age should be managed by experts in paediatric HTN and it is a paediatrician or a physician with experience treating children with HTN who should initiate the treatment. In children, medications need to be dosed on a milligramme per kilogramme basis and dosing ranges might differ from those in adults because of differences in drug metabolism and body composition (7). Two only drugs with paediatric liquid formulations: valsartan (3 mg/ml) and acebutolol (40 mg/ml) are approved by European health authorities and available in private pharmacy for paediatric hypertension. They can be useful in younger children.

The European Medical Agency (EMA) has promoted an investigation paediatric plan to improve evidences-based medicine in paediatric drugs but not all classes of drugs are studied in children.

We should keep in mind that the benefits and harms of long-term pharmacologic treatment is not known as trial duration is generally limited to 2 to 4 weeks. Several randomised controlled trials of ACE inhibitors (among them enalapril, lisinopril) and of ARBs (losartan, valsartan, candesartan) in children with HTN have shown a good BP response to the medication (57–62). Safety and tolerability of valsartan in children 6 to 17 years of age with HTN have been recently evaluated by EMA and by the French *Haute Autorité de Santé* (HAS) (63, 64). In children with HTN and renal failure, proteinuria, or diabetes mellitus, an ACE inhibitor or ARB is recommended as the initial antihypertensive agent unless there is an absolute contraindication (9). ACEi and ARBs are contraindicated during pregnancy and warrant contraception/abstinence in female patients of child-bearing age and alternative medications (e.g., calcium channel blocker, β-blocker) can be considered when appropriate (9). Caution should be exercised in situations where there is a risk of dehydration (acute gastroenteritis), especially in younger patients receiving ACEi or ARBs, and laboratory tests should be performed in any doubt. The concomitant use of racecadotril, indicated for acute diarrhoea, and ACEi may increase the risk of angioedema. Therefore, the benefit/risk balance should be carefully assessed before initiating racecadotril treatment in patients taking ACEi.

Other medications studied for paediatric HTN have less consistent results. Trials of the long-acting dihydropyridine calcium channel blockers (amlodipine and felodipine) have shown for amlodipine a difference from placebo in SBP response vs. placebo in 268 children aged from 6 to 16 years (65, 66). ACEi, ARBs, and calcium channel blockers reduced BP similarly (67).

Paediatric experience for thiazide diuretics has been reported but data about their efficacy are missing on monotherapy; Loop diuretics are used in case of cardiac or renal failure but unlike in adult medicine, they are very rarely used for HTN in children even if they are included in the US recommendations in first line therapy or in combination (9). According to US guidelines, beta-blockers are less well-studied in children with HTN (except metoprolol) (68) and are not recommended for first-line treatment, due to the potential side effect profile (9).

Table 5 below lists the main drug classes used for the chronic treatment of HTN in children. The prescribing principles for these therapeutic classes in children and adolescents are the same as for adults. Pharmacologic treatment of HTN should be initiated with an ACEi, ARB, long-acting calcium channel blocker, or a thiazide diuretic (even if in France, thiazide diuretics are very rarely used) (9). The use of a combination product as initial treatment has not been studied and cannot be recommended in children. For good treatment compliance, monotherapy should be favoured with as few as possible daily doses, at times compatible with family life. The choice of the initial molecule should be guided by the aetiology and initiated at the lowest dosage strength, then gradually increased to reach the target BP. If BP is not controlled with the highest dose of the treatment, a 2nd molecule should be introduced with, if possible, rapid switching to fixed-dose combination therapy to promote compliance. The reader should know that data on combined therapies in children are scarce (69), and after initiation of treatment, BP should be checked every 2 to 4 weeks until the HTN is under control, then every 3 to 4 months.

**Table 5:** Main drug classes that can be used in children.

**Class**	**Drug**	**Initial dose**	**Dose per 24 h**	**No. doses/24 h**	**Undesirable effects, precautions**
Calcium channel blockers	Amlodipine	0.1–0.2 mg/kg Max 5 mg	0.6 mg/kg/d Max 10 mg/d	1	Preparation of a 1 mg/mL suspension of amlodipine otherwise tablets of 2.5, 5 or 10 mg Tachycardia, flushing, headache, possible peripheral oedema, gingival hypertrophy
	Nicardipine LP	0.25–0.5 mg/kg/d	1–3 mg/kg/d Max 120 mg/d	2	
	Felodipine	5 mg	10 mg	1	
ACE inhibitors	Captopril	Liquid formulation: 5 mg/5 ml and 25 mg/5 ml Newborns: 0.01–0.03 mg/kg/dose Infants: 0.1 mg/kg/dose Older children: 0.3–0.5 mg/kg/dose	6 mg/kg/d Max 150 mg	1 to 3	Monitoring of electrolytes after introduction or increase in dosage (hyperkalaemia, kidney failure) Cough and angioedema under ACE inhibitors Contraindicated during pregnancy (fetotoxicity) Treatment should be interrupted in case of gastroenteritis/dehydration
	Enalapril	Newborns: 0.05 mg/kg/dose Infants: 0.05 mg/kg/dose Older children: 0.08 mg/kg/dose max 5 mg	0.8 mg/kg/d Max 40 mg/d	1	
	Lisinopril	0.08 mg/kg Max 5 mg/d	0.6 mg/kg/d Max 40 mg/d	1	
ARBs	Losartan^*^	0.7 mg/kg/d Max 50 mg/d	1.4 mg/kg/d Max 100 mg/d	1	
	Irbesartan	2 mg/kg/d	6–12 years <35 kg: 75–150 mg/d ≥13 years >35 kg: 150–300 mg/d	1	
	Valsartan	3 mg/ml drug with liquid formulation	– 1–6 years: 1 mg/kg/d and <4 mg/kg/d – 6–18 years: <35 kg: 20 mg/d and <40 mg/ d >35 kg: 40 mg/d and <80 mg/d		
Beta blockers	Acebutolol	1.5–3 mg/kg/d	5–15 mg/kg/d	1 to 2	Contraindications: AV block not cardioselective (Propranolol); contraindicated in case of asthma and heart failure Limit certain athletic performances
	Acebutolol	40 mg/ml in liquid formulation	10–20 mg/kg/d	1 to 2	
	Propranolol	1 mg/kg/d	4 mg/kg/d Max 640 mg/d	2 to 3	
	Atenolol	0.1–1 mg/kg/d	2 mg/kg/d Max 100 mg/d	1 to 2	
Alpha and beta blocker	Labetolol	1–3 mg/kg/d	10–15 mg/kg/d Max 1,200 mg/d	2	Contraindications: AV block, asthma, heart failure Limit certain athletic performances
Alpha blockers	Prazosin	0.05–0.1 mg/kg/d Max 0.5 mg × 2 per d	0.5 mg/kg/d Max 20 mg per d	2 to 3	Risk of orthostatic hypotension after the 1st dose Fatigue, concentration difficulties
	Clonidine	5 μg/kg/d	30 μg/kg/d max 1.05 mg/d	2 to 3	
Diuretics	Hydrochlorothiazide	0.5–1 mg/kg/d	3 mg/kg/d Max 50 mg/d	1	Monitoring of electrolytes Furosemide is useful for the complementary treatment of HTN in case of kidney failure
	Furosemide	0.5–2 mg/kg/dose	6 mg/kg/d	1 to 2	
	Spironolactone^*^	1 mg/kg/d	3.3 mg/kg/d Max 100 mg/d	1 to 2	

* *Tablets can be crushed doses are given as examples, and the prescription of the drug should remain the physician's own responsibility*.

### Recommendation No. 8

a) We recommend a change in lifestyle and dietary habits in all cases of childhood HTN (Grade C, class 1).
b) We recommend that a paediatrician or a physician with experience in the treatment of HTN in children and adolescents initiates it (Grade C, class 1).
c) Pharmacological treatment should be initiated in cases of (Grade A, class 1):
  * Symptomatic or stage 2 HTN
  * Secondary HTN
  * Damage to target organs (heart, eyes)
  * Kidney failure
  * Concomitant type 1 or type 2 diabetes
  * Persistence of HTN despite a change in lifestyle and dietary habits, regardless of the cause as the symptoms have an impact on target organs.
d) In children, we recommend to use long-acting calcium channel blockers or angiotensin-converting-enzyme inhibitors (ACEi) / angiotensin receptor blockers (ARBs) (Grade B, class 1).
e) We recommend to target a BP under the 75th age percentile, and even below the 50th percentile in cases of kidney failure and/or concomitant proteinuria (Grade B, class 1).

### Special Cases

#### Hypertensive Emergency: From Which Value and What to Do

Chronic untreated HTN can cause growth retardation, so it is essential to follow growth on a dedicated growth curve. Clinically, HTN may manifest as headaches, epistaxis, ringing in the ears, and malaise during physical exertion. Severe HTN can cause abdominal pain, vomiting, anorexia, a break in the growth curve, polydipsia and polyuria, and recurrent peripheral facial paralysis. Hospitalisation in a specialised paediatric unit is required in the event of symptomatic HTN for rapid institution of treatment (and an aetiological assessment).

HTN may also be revealed by a complication: heart failure with acute pulmonary oedema, reduced visual acuity, convulsions, coma and, possibly, signs of vascular insufficiency resulting in oedema and/or cerebral haemorrhage. In new-borns (0–1 months) and infants (1–12 months), HTN may present as vasomotor disturbances or malaise, but is most often revealed by heart failure. In such cases, the condition may be life-threatening due to multiple organ or heart failure, or hypertensive encephalopathy.

Malignant HTN is a life-threatening emergency and includes HTN with organ dysfunction, i.e., neurological, renal or cardiac impairment. Children presenting a hypertensive emergency should be taken care of in a continuing care/intensive care unit so they can be closely monitored and receive organ support. It is recommended to lower BP by 25% in the first 6–8 h, then more gradually over the following 24–48 h. Too rapid normalisation of BP can lead to more serious side effects than the HTN itself.

The reference treatment is intravenous treatment with continuous modes being preferred to boluses to avoid arterial hypotension (risk of organ hypoperfusion and neurological sequelae such as loss of vision). Nicardipine (calcium channel blocker, vasodilator) is the first-line treatment because it allows gradual and controlled decrease in BP with no risk of sudden hypotension. In addition, it is one of the only emergency treatments that is not contraindicated in case of stenosis (renal, carotid or cerebral arteries). The treatment permits a gradual and adjustable decrease in BP. Labetalol (alpha and beta blocker) can also be used in hypertensive emergencies. Diuretics (furosemide and bumetanide) are indicated in hypertensive emergencies with volume overload (Table 6). In all cases, the patient must be managed in a specialised paediatric setting, possibly after transfer by the emergency medical service (SAM) to a dedicated unit.

**Table 6:** Management of hypertensive emergencies.

**Drug (brand name)**	**Therapeutic class**	**Administration route**	**Dosage**	**Time to effect**	**Duration of effect**	**Contraindications**	**Side effects**
Nicardipine (Loxen)	Calcium channel blocker	IV	1–3 μg/kg per min	A few minutes	30 to 60 min	/	Reflex tachycardia Headache, flushing, nausea, inflammation at the injection site
Labetolol (Trandate)	Alpha and beta blocker	IV	2–20 mg/kg/day	5 to 10 min	3 to 24 h	Heart failure, Atrioventricular block, Asthma	Bradycardia, hypotension, nausea
Furosemide (Lasilix)	Loop-acting diuretic	IV, IM, or PO	Slow IV 30 min 0.5 to 3 mg/kg/dose every 3 to 4 h. Up to 10 mg/kg/day	5 min	2 to 3 h		Hypokalaemia Ototoxicity Increased blood glucose levels
Bumetanide (Burinex)	Diuretic	IV	0.02 mg/kg/injection up to 1 mg/kg/day	5 min	2 to 3 h		Hypokalaemia Increased blood glucose levels

*Doses are given as examples, and the prescription of the drug should remain the physician's own responsibility*.

### Recommendation No. 9

We recommend that children presenting with a hypertensive emergency be managed in a specialised continuous care/intensive care unit with paediatric experience (Grade A, class 1).

#### Hypertension and Contraception in Adolescents

The various methods of contraception and the time required to implement contraception in adolescents, detailed in other works (70, 71), will not be addressed in this paper. However, two separate situations can be distinguished: onset of HTN in an adolescent using a combined hormonal contraceptive and contraception prescription in hypertensive adolescents.

#### Onset of Hypertension in an Adolescent Using a Combined Hormonal Contraceptive (CHC)

The literature presents no arguments for the prescription of a specific type of contraception in adolescents except in the event of definite contraindications. Combined hormonal contraceptives (CHC) and condoms are by far the methods the most used by adolescent girls.

HTN can occur regardless of how long the CHC has been taken and its route of administration. It depends on the dose of ethinylestradiol (EE), especially for the high-dose pills (50 μg EE). Its severity varies, ranging from mild HTN to malignant HTN. Its frequency of onset after the introduction of a CHC is between 0.6 and 8.5% (72).

In the context of use of a CHC, onset of HTN can be explained by increased production of liver angiotensinogen and by water and sodium retention following an action on the mineralocorticoid receptors. CHC modify endocrine parameters (plasma renin activity and aldosterone levels) for 4 to 6 weeks.

Factors that predispose adolescents using CHCs to HTN include being overweight and obesity as well as a family history of HTN (73–75).

In adolescents, as in older women, it is essential to measure BP along with weight and BMI before the first initiation of a CHC. These measurements should be repeated at each follow-up visit to screen for asymptomatic HTN. In addition, patients suffering from with headaches, whether migraine or non-migrainous headaches, should be screened for HTN during visits and on an outpatient basis, especially overweight, obese or diabetic adolescents (masked HTN) (74–76).

The type of CHCs that should be prescribed first-line to adolescents choosing this type of birth control are so-called second generation CHCs (containing 20 or 30 μg of EE and levonorgestrel). None of the other CHCs (except for the one containing norgestimate) should be used first-line due to the higher risk of venous thromboembolism as compared with second generation CHCs both in adolescents and older women. When the CHC is prescribed, the patient should also be advised to use condoms for effective prevention of sexually transmitted infections.

Blood pressure should be reassessed at 3, 6 months, and then every year. If a significant increase in BP is observed during a visit (>140/90 mm Hg), HBPM or 24 h ABPM should be implemented to avoid an alarm reaction and to rule out a white coat effect. In the event of HTN confirmed during a visit, the CHC should be discontinued and replaced by a so-called progestin-only contraceptive (containing only small doses of progestins and that may be administered by various routes [oral route (pill), subcutaneous route (implant), intrauterine route (IUD)]. The effect of progestin-only contraceptives on BP appears to be neutral. If BP doesn't drop to normal levels within 3 months, the cause of the HTN must be investigated and pharmacological treatment initiated along with lifestyle changes.

It should be noted that in adolescents (as in older women), first-line prescription of CHCs delivered by alternative routes [transdermal (patch) or vaginal (ring)] should be avoided as they contain 3rd generation progestins which are associated with a higher thrombogenic risk as compared with second generation CHCs and also expose patients to an increase in BP. The alternative transdermal and vaginal administration routes should not be used to replace an orally administered CHC: in the event of HTN with a CHC pill requiring interruption of this birth control method, it is not recommended to replace the pill with a CHC delivered by an alternative route.

### Recommendation No. 10

a) We recommend measuring BP at the initiation of CHC treatment, then periodically, at 3 and 6 months, and then annually during follow-up visits (Grade B, class 1).
b) We recommend measuring BP in patients presenting with headaches, whether migraine headaches or not, at the initiation of CHC treatment to screen for HTN (Grade B, class 1).
c) In case of confirmed HTN or stage 3 HTN during a visit (>180/110 mm Hg), we recommend to replace the CHC by a progestin-only birth control method (pill, implant, or IUD) (Grade A, class 1).
d) In adolescents, we suggest to avoid the first-line prescription of CHCs delivered by alternative routes [transdermal (patch) or vaginal (ring)] (Grade C, class 2).
e) When an oral contraceptive is prescribed, patients should be advised to also use condoms (Grade C, class 2).

#### Contraception Prescription in Adolescents With Hypertension

Use of a CHC in hypertensive patients leads to an increase in systolic and diastolic BP, while discontinuation is associated with a significant drop in these values (72). Thus, in the SPCs of CHCs, their prescription is absolutely contraindicated in the event of essential or secondary HTN. The risk related to the use of CHCs is indeed unacceptable and their prescription is contraindicated in stage 2 or 3 HTN, HTN complicated by target organ damage, or HTN associated with other uncontrolled CV risk factors (obesity, diabetes, smoking, etc.). Consequently, the French HAS recommends avoiding the use of CHCs in hypertensive patients and contraindicates their use in stage 2 or 3 HTN or in case of concomitant cardiovascular disorders (77).

The French Society of Hypertension has developed an expert consensus decision pathway on “HTN, Hormones and women/GRADE method,” but it does not address the specific case of adolescents (78). The consensus recommends not prescribing a CHC to hypertensive women (controlled or uncontrolled), regardless of the route of administration (oral route, transdermal patch, vaginal ring), because these forms of contraception are associated with a risk of an increase in BP; it is recommended to prescribe hypertensive women seeking a birth control method with an effective form of contraception, i.e., a progestin-only contraceptive regardless of the route of administration (oral, subcutaneous or intrauterine route), or a copper IU, providing there are no gynaecological contraindications.

Finally, if the adolescent prefers a copper IUD, it is possible to implant the devices in adolescents, providing certain precautions are taken and the adolescent is fully informed about the device before its implantation (70). Not having had a child is not a barrier to the use of this type of birth control.

In hypertensive adolescents with incurable causes of HTN, the group proposes not to prescribe any form of CHC and to use only the options applicable to hypertensive women.

When adolescents are prescribed with contraceptives, they should also be advised to use condoms for effective prevention of sexually transmitted infections.

It should be noted that regardless of the type of antihypertensive treatment taken, progestin-only contraceptives pose no drug-drug interaction problems, contrary to certain other treatments (antiepileptics for example).

Furthermore, it is important to ensure that the methods of contraception used by hypertensive adolescents are very effective to avoid unwanted pregnancies. Practitioners need to remind an adolescent girl of childbearing age that ACEi/ARBs are contraindicated during pregnancy and warrant contraception. The patients also need to be informed that they will need to plan any future pregnancy because their antihypertensive treatment may need to be modified as some treatments are incompatible with pregnancy (78).

### Recommendation No. 11

a) We recommend not to prescribe combined hormonal contraceptives, regardless of the route of administration (oral, vaginal or transdermal), to adolescents with uncomplicated mild HTN or severe stage 2 or 3 HTN that may/may not be complicated by target organ damage and/or concomitant cardiovascular disease (Grade B, class 1).
b) We recommend to offer hypertensive adolescents an effective progestin-only contraceptive that can be administered by various routes (oral, subcutaneous or intrauterine routes) or a copper IUD, providing there are no gynaecological contraindications (Grade C, class 2).

## Conclusion

Hypertension in children and adolescents, which is less common than in adults, requires the involvement of several medical professionals including paediatricians as the definition of childhood HTN is very different from that of adult HTN. Confirming a diagnosis of HTN is not easy, particularly in childhood. After a first diagnosis of HTN, generally made by the attending physician or paediatrician, it is suggested that children and adolescents be more specifically referred to a paediatric or adult nephrologist, cardiologist or endocrinologist for further examinations and treatment. In this manuscript, we propose clinical practise points to help general physicians and paediatricians to improve the diagnosis and general management of paediatric HTN in daily practise, as summarised in Table 7.

**Table 7:** Summary of the statements.

Definition of HTN in children and adolescents	To define HTN, we recommend using the normative BP tables developed according to age, height and gender, a simplified version of which is provided in Table 1 (Grade C class 1)
Epidemiology	a) Before the age of 3 years, we recommend to measure BP systematically in the following cases: - History of low birth weight <2,500 grams; - Kidney disease or urological abnormality; - Congenital heart disease; - Solid organ or bone marrow transplantation; - Intracranial HTN; - Exposure to medicines known to cause HTN; - Systemic disease which may be complicated by HTN (neurofibromatosis, tuberous sclerosis, etc.) (Grade C class 1).
	b) After the age of 3 years, we recommend to measure BP systematically at least once a year in the same way as weight, height and BMI as HTN is most often asymptomatic (Grade C class 1)
Method of BP measurement	a) In children, we recommend to measure BP using an auscultatory method and an aneroid sphygmomanometer (Grade C, class 1) b) In the event of an abnormal BP reading with an oscillometric BP monitor (which tends to overestimate BP), we recommend to check the measurement using an auscultatory manual method (Grade C, class 1) c) We recommend to measure BP in a calm place in a child who has been sitting for 5 min with his/her feet on the floor (not suspended), whose back and arms are supported, and whose antecubital fossa is at heart level. An appropriately sized cuff and a BP monitor that has been validated in children must be used (Grade C, class 1) d) We recommend to measure BP in the right arm (site spared in case of coarctation of the aorta) (Grade C, class 1) e) In the event of elevated BP during a 1st visit, we recommend in asymptomatic patients to repeat measurements during 2 other visits 1 month apart (Grade C, class 1) or closer together in high-risk cases (Grade B, class 1) f) 24 h Ambulatory Blood Pressure Monitoring (ABPM) can be performed in selected situations by experts in paediatric HTN, on a case-by-case basis (Grade B, class 1) for children >120 cm in height, but it may not be well-tolerated; ABPM is not recommended in children who measure <120 cm g) We recommend to only use Home Blood Pressure Monitoring (HBPM) for the monitoring of known HTN (Grade C, class 1) given the lack of reference values for the diagnosis of HTN
Clinical examination	In children and adolescents being evaluated for high BP, the practitioner should perform a physical examination to identify findings suggestive of secondary causes of HTN listed in Table 3 (Grade B, class 1)
Additional tests	a) We recommend to perform the following tests in all children and adolescents regardless of the results of the clinical examinations: blood electrolytes (serum potassium), serum creatinine, assessment of glomerular filtration (using the Schwartz formula in children), urine sediment examination of the first morning urine (haematuria), urine protein to creatinine ratio (normal <50 mg/mmol before 2 years of age and <20 mg/mmol after 2 years of age) (Grade A, class 1) b) The following tests should also be requested in overweight or obese children or adolescents (BMI >95th percentile) and in those with a family history of dyslipidaemia: fasting blood sugar, fasting lipid profile including total cholesterol, HDL and LDL, triglycerides, AST, and ALT (Grade A, class 1) c) Once these initial examinations have been requested, we recommend to seek the opinion of a paediatric cardiologist and/or nephrologist and/or endocrinologist (Grade C, class 1) d) We recommend to seek the expertise of a skilled paediatric cardiologist to interpret ECGs in children under 12 years of age (Grade B, class 1) e) We recommend to systematically perform cardiac echocardiography to screen for LVH and isthmic coarctation of the aorta (Grade A, class 1) f) We recommend to systematically perform Doppler ultrasound and kidney ultrasound to determine whether HTN can be attributed to a renal cause (asymmetry, renal hypoplasia) (Grade A, class 1)
Secondary causes of HTN	a) Regardless of the age of the child, we recommend to carefully check for a secondary cause of HTN (Grade B, class 1) b) We suggest focusing the search for secondary HTN on renal or cardiac causes as they account for 2/3 of the causes of secondary HTN (Grade B, class 2)
Healthcare pathway	a) After a first diagnosis of HTN generally made by the attending physician or the paediatrician, we suggest to refer children or adolescents more specifically to a paediatric nephrologist or nephrologist when they present with (Grade C, class 2): - Family history of kidney disease (kidney failure, renovascular dysplasia, acute pyelonephritis); - History of uropathy, tubulopathy; - Kidney failure, urine sediment disorders; proteinuria - Vascular murmur in the renal area; - Asymmetric kidney size or a single kidney b) We suggest to refer children or adolescents more specifically to a paediatric cardiologist or cardiologist when they present with (Grade B, class 2): - Clinical signs such as tachycardia, malaise, or heart murmur on auscultation; - Absence or weak pulse in the legs which suggests aortic coarctation; - Family history of heart disease; - Williams-Beuren syndrome or Turner or Alagille syndromes which predispose to the risk of aortic coarctation
	c) We suggest to refer children or adolescents more specifically to a paediatric endocrinologist or endocrinologist when they present with (Grade B, class 2): - Clinical signs of hypercortisolism (weight gain and growth failure, facial and truncal obesity, proximal amyotrophy, vertical purple stretch marks, facial erythrosis +/– hirsutism); - Short stature (Turner syndrome) or abnormal tallness (acromegaly); - Goitre; - Severe obesity
Principles of therapeutic strategy	a) We recommend a change in lifestyle and dietary habits in all cases of childhood HTN (Grade C, class 1) b) We recommend that a paediatrician or a physician with experience in the treatment of HTN in children and adolescents initiates it (Grade C, class 1) c) Pharmacological treatment should be initiated in cases of (Grade A, class 1): * Symptomatic or stage 2 HTN * Secondary HTN * Damage to target organs (heart, eyes) * Kidney failure * Concomitant type 1 or type 2 diabetes * Persistence of HTN despite a change in lifestyle and dietary habits, regardless of the cause as the symptoms have an impact on target organs
	d) In children, we recommend to use long-acting calcium channel blockers or angiotensin-converting-enzyme inhibitors (ACEi)/angiotensin receptor blockers (ARBs) (Grade B, class 1) e) We recommend to target a BP under the 75th age percentile, and even below the 50th percentile in cases of kidney failure and/or concomitant proteinuria (Grade B, class 1)
Special cases	We recommend that children presenting with a hypertensive emergency be managed in a specialised continuous care/intensive care unit with paediatric experience (Grade A, class 1)
Contraception in teenagers	a) We recommend measuring BP at the initiation of CHC treatment, then periodically, at 3 months and 6 months, and then annually during follow-up visits (Grade B, class 1) b) We recommend measuring BP in patients presenting with headaches, whether migraine headaches or not, at the initiation of CHC treatment to screen for HTN (Grade B, class 1) c) In case of confirmed HTN or stage 3 HTN during a visit (>180/110 mm Hg), we recommend to replace the CHC by a progestin-only birth control method (pill, implant, or IUD) (Grade A, class 1) d) In adolescents, we suggest to avoid the first-line prescription of CHCs delivered by alternative routes [transdermal (patch) or vaginal (ring)] (Grade C, class 2) e) When an oral contraceptive is prescribed, patients should be advised to also use condoms (Grade C, class 2)
Contraception in adolescents with HTN	a) We recommend not to prescribe combined hormonal contraceptives, regardless of the route of administration (oral, vaginal or transdermal), to adolescents with uncomplicated mild HTN or severe stage 2 or 3 HTN that may/may not be complicated by target organ damage and/or concomitant cardiovascular disease (Grade B, class 1) b) We recommend to offer hypertensive adolescents an effective progestin-only contraceptive that can be administered by various routes (oral, subcutaneous or intrauterine routes) or a copper IUD, providing there are no gynaecological contraindications (Grade C, class 2)

## Data Availability Statement

The original contributions presented in the study are included in the article/supplementary material, further inquiries can be directed to the corresponding author/s.

## Author Contributions

BB coordinated the activities of the group, the preparation of the article, and its submission. BB, PS, KB, CM-V, GP-B, and JB drafted the manuscript. SH, CP, BR, LM, JMB, and JF critically revised the manuscript. All authors approved the final article.

## Group of Readers

From the French Society of Hypertension: Laurence Amar; Theodora Angoulvant; Michel Azizi; Jacques Blacher; Romain Boulestreau; Pierre-Yves Courand; Thierry Denolle; Pierre Fesler; Xavier Girerd; Jean-Michel Halimi; Olivier Hanon; Pierre Lantelme; Sylvain Le Jeune; Benoît Lequeux; Marilucy Lopez-Sublet; Jean Jacques Mourad; Atul Pathak; François Silhol; Pierre-Louis Tharaux; Emmanuelle Vidal Petiot.

## Conflict of Interest

JMB was employed by the company Inserm CIC-P Pierre Drouin, France. The remaining authors declare that the research was conducted in the absence of any commercial or financial relationships that could be construed as a potential conflict of interest.
